# A Review: Origins of the Dielectric Properties of Proteins and Potential Development as Bio-Sensors

**DOI:** 10.3390/s16081232

**Published:** 2016-08-04

**Authors:** Fabien Bibi, Maud Villain, Carole Guillaume, Brice Sorli, Nathalie Gontard

**Affiliations:** 1 Joint Research Unit Agropolymers Engineering and Emerging Technologies, UMR 1208 INRA/SupAgroM/UMII/CIRAD, 2 Place Pierre Viala, Montpellier 34060, France; bibi.fabien@hotmail.com (F.B.); maud.villain@unistra.fr (M.V.); c-guillaume@univ-montp2.fr (C.G.); gontard@univ-montp2.fr (N.G.); 2 B. SORLI, Institut d’Electronique et des Systèmes, Université Montpellier 2, 860 Rue Saint-Priest, Montpellier 34090, France

**Keywords:** dielectric permittivity and loss, bio-sensor, proteins, dielectric and structure modification, vapors and gases, physico-chemical properties

## Abstract

Polymers can be classified as synthetic polymers and natural polymers, and are often characterized by their most typical functions namely their high mechanical resistivity, electrical conductivity and dielectric properties. This bibliography report consists in: (i) Defining the origins of the dielectric properties of natural polymers by reviewing proteins. Despite their complex molecular chains, proteins present several points of interest, particularly, their charge content conferring their electrical and dielectric properties; (ii) Identifying factors influencing the dielectric properties of protein films. The effects of vapors and gases such as water vapor, oxygen, carbon dioxide, ammonia and ethanol on the dielectric properties are put forward; (iii) Finally, potential development of protein films as bio-sensors coated on electronic devices for detection of environmental changes particularly humidity or carbon dioxide content in relation with dielectric properties variations are discussed. As the study of the dielectric properties implies imposing an electric field to the material, it was necessary to evaluate the impact of frequency on the polymers and subsequently on their structure. Characterization techniques, on the one hand dielectric spectroscopy devoted for the determination of the glass transition temperature among others, and on the other hand other techniques such as infra-red spectroscopy for structure characterization as a function of moisture content for instance are also introduced.

## 1. Introduction

It is widely recognized that dielectric properties play an important role in assessing the functions of proteins. Indeed, considerable attention is continuously being given for years [1,2,3,4,5,6] to study the variation of proteins properties, particularly mechanical, electrical, dielectric and structural properties. Electrostatic interactions are important for the global stability of the protein structure [2,6], and are considered to be the sum of contributions from van der Waals forces, hydrogen bonds, covalent bonds, and hydrophobic interactions [1].

Owing to the aforementioned interactions, bonds not only stabilize the protein structure but also contribute to the electrical and dielectric properties of the proteins, themselves playing a crucial role in the stability of the structure [7]. These give rise to interactions or polarizations, namely interfacial polarization, strong dipole-dipole interactions which is the sharing of electron pairs between atoms (stable electronic configuration), atomic interactions and electronic interactions [8]. Consequently, proteins are considered as polarizable materials, which can be characterized by dielectric spectroscopy [9]. Frequency is another critical factor influencing the behavior of dielectric materials such as proteins, particularly their polarization. With increasing frequency, polarizations will no longer be able to follow the quick electric field reversals, resulting in the dropping out of constituents, while others still prevail [8,10,11]. This results in a drop of the dielectric permittivity and dielectric loss values of the protein. Bonincontro and Risuleo [12] suggest that protein solutions exposed to an electric field in the radio frequency range (3 kHz to 300 GHz) generally show dielectric relaxation due to orientation polarization (dipole-dipole interactions). The authors are able to determine two significant parameters (the effective hydrodynamic radius and the electric dipole moment) from the dispersion curve, characterizing conformation and structure of the protein.

Although several reports exist on protein sensitivity in contact with water vapor, oxygen, carbon dioxide [13,14] and ethanol gas [15,16,17], supplementary knowledge on their effects on structural and dielectric behaviors of proteins is lacking in literature. Furthermore, regarding their electrical properties, their dielectric properties, and their sensitivity to environmental factors such as water vapor and carbon dioxide, it is hypothesized that proteins can be used as bio-sensors for the measurement of a physical, chemical, biological or any other parameter, giving a response in terms of resistance, voltage or current [18]. Several parameters should also be studied in the development of the bio-sensors such as: the measurement range on which the bio-sensor is sensitive to analytes, the accuracy and precision, the stability of measurements, and the repeatability of measurements.

According to literature, some bio-sensors have been developed by performing molecular imprint of the sensing material able to recognize analytes, thus having a high selectivity. Consequently, a particular molecule can be identified due to the shape of the imprinted cavity specific to the molecule to be detected. This was performed in the objective of detecting meat spoilage through color changes [19,20]. Other studies have been performed on the use of proteins as switches [21], as well as on the sensing principle of proteins by color changes when detection takes place [22,23]. With respect to this, it could be interesting to consider another sensing ability, making use of plant polymers, for instance proteins, based on their electrical and dielectric properties, and on their sensitivity to environmental conditions.

The present review aims to an in-depth analysis of the dielectric properties of proteins and to identify the factors influencing their properties. The interest raised by proteins resides in the possibility to use the dielectric permittivity and loss to assess the changes in the structural conformation at high frequency. To a further extent, a detailed analysis of these characteristics (electric and dielectric properties, sensitivity to vapors and gases, frequency, sensor parameters) would lead to significant findings regarding the potential development of bio-sensors with proteins films, as innovative eco-friendly detection method, for applications such as intelligent packaging systems to monitor food packed products.

## 2. Dielectric Properties of Polymers

### 2.1. Origins of Dielectric Properties of Polymeric Materials

The response of a material to an applied electric field is described by its conductivity and permittivity. The latter is often expressed as the complex permittivity (ε*) composed of the dielectric permittivity (real part) and the dielectric loss (imaginary part), represented by Equation (1). The dielectric permittivity (ε′) can be defined as the ability of the material to store energy, and the dielectric loss factor (ε″) is its ability to dissipate energy into heat by frictional motion of the elements carrying chargers [24]. According to Castro-Giraldez et al. [25], dielectric loss can also be due to ionic conductivity which is introduced into the material when exposed to an electromagnetic field, e.g., in the presence of salts at a high hydration level. Both dielectric loss and permittivity constitute the dielectric character of the material.

(1) $$\varepsilon^{*}=\varepsilon^{\prime }-j\varepsilon^{\prime \prime }$$

**Figure 1:**
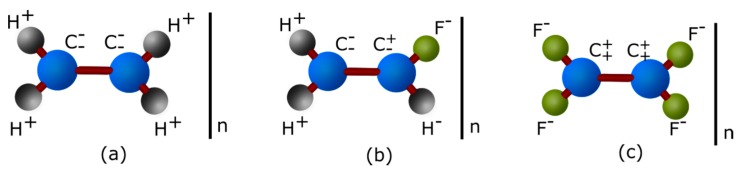
Polyethylene apolar molecule (**a**); poly(fluoroethylene) polar molecule (**b**); and poly(tetrafluoroethylene) apolar molecule because of its symmetry (**c**).

Polymers can be classified as polar or apolar (Figure 1) depending on their chemical structure and their permittivity evolution with frequency and temperature [26]. Apolar polymers are usually efficient isolating materials with a permittivity loss around 10^−4^ to 10^−3^ and a quasi-constant permittivity of 2.5 to 3, in a broad range of frequencies and temperatures. Most of them are mainly composed of carbon and hydrogen atoms symmetrically disposed all along a carbon chain. As examples, polyethylene and poly(tetrafluoroethylene) are apolar polymers.

Polar polymers are composed of molecules containing electronegative atoms e.g., nitrogen, oxygen, chloride and fluoride providing dipoles to the polymer. Higher dielectric constant (3.5 to 10) and loss factor (10^−2^ to 10) characterize them. Proteins form part of the polar polymer group even if the polar character is more or less strong depending on the lateral chain of amino acids present.

**Figure 2:**
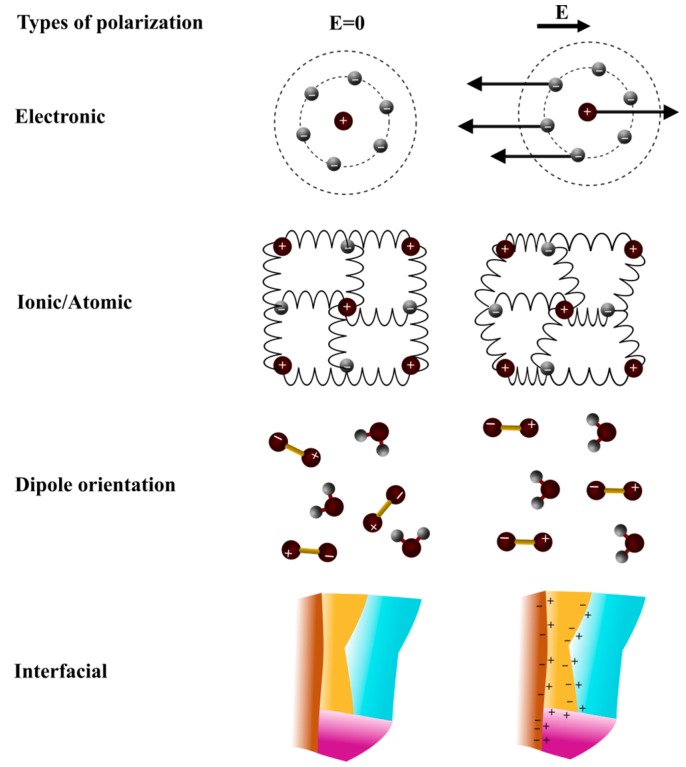
Schematic representation of four types of polarization [32].

Polarization of material components is due to the modification of electric charges repartition when an electric field is applied. In an alternative electric field, polarization does not follow immediately the electric field switch. A response time or delay exists corresponding to the dielectric relaxation, inducing an energy loss. Polarization can be due to various effects, ranging from charge accumulation at surfaces of materials having layers of different permittivity values (interfacial polarization), to dipole orientation, up to atomic and electronic polarization (Figure 2) [27,28]. Each of these polarization effects contribute to the dielectric constant global value and are responsible for: relaxation phenomena taking place up to the THz, resonance phenomena happening at frequencies above the THz.

**Figure 3:**
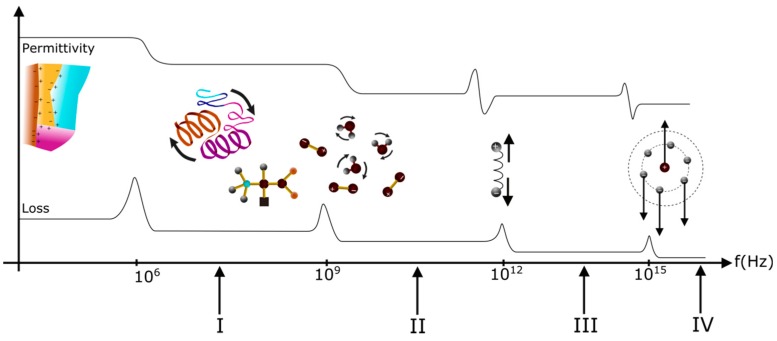
Influence of different types of polarization on dielectric parameters: I, radio frequency range; II, microwave frequency range; III, infrared-visible frequency range; and IV, ultraviolet frequency range.

Dielectric permittivity and dielectric loss phenomena can be schematically represented (Figure 3) on a wide range of frequency.

Considering Figure 3, 10^6^–10^10^ Hz is the frequency range of interest of some authors [29,30,31]. In this range, the secondary structure as well as dipoles found within the protein will be impacted by the effects of the electric field frequency. Depending on the material structure and interaction forces, i.e., van der Waals forces, hydrogen bonds, covalent bonds, and hydrophobic interactions, the impact of the electric field may vary, particularly on the protein relaxation dynamic, thus imposing the dielectric values. With increase in frequency, the secondary structure is less impacted by the electric field, having a more considerable effect on smaller elements in the network such as dipoles. Referring to Figure 3, the dielectric permittivity value imposed is then inferior to the permittivity value recorded for the broad secondary structure, which is impacted at lower frequency.

Considering Figure 2 and Figure 3, four types of polarization are presented. At low frequency, they contribute to the high value of the dielectric constants. When frequency rises, contributions brought by each type of polarization phenomena disappear, followed by a decrease in the dielectric constants.

The first kind of polarization named interfacial polarization, so-called Maxwel-Wagner effect, appears at low frequencies (<10^6^ Hz). This type of polarization occurs in heterogeneous material. It comes from the limited displacement of free charges and their accumulation at interfacial areas or borders between the different phases constituting the material when these different phases possess distinct permittivity values. This phenomenon is specific for solid and liquid dielectrics, especially with non-homogeneous or amorphous structures.

The dipole orientation also called Debye polarization implies the orientation of polar molecules under an electric field at higher frequency (10^6^ to 10^10^ Hz). This type of polarization is mainly due to the rotation of amino acids, rotation of charged side groups of proteins, and the relaxation of water interacting with proteins. These molecules are of asymmetric type, where the gravity center resulting from negative charges of such a molecule does not match with the one of positive charges. Such molecules are electric dipoles and usually have a permittivity value of 4 or 5 at low hydration level, and up to 10 at higher hydration level. In addition, the loss values usually obtained are low, in the order of magnitude of 10^−3^ to 10^−2^, which vary with temperature, relative humidity and frequency. With the application of an alternating electric field and due to the viscosity of the medium, molecules cannot orientate themselves instantaneously as they are submitted to Debye forces, such as viscosity forces. Variations of ε′ and ε″ with frequency are called dispersion and absorption curves, respectively.

Atomic polarization (or ionic) is a result of the atoms displacement linked via ionic bonds (10^10^ to 10^13^ Hz). Valence electrons travel around orbits shared with others atoms thus creating an induced dipole. This induced dipole sticks to the electromagnetic field orientation, but also takes into account relaxation dynamics. Ionic bonds are found in inorganic solid dielectric materials with a crystalline structure, e.g., ceramic materials. This kind of polarization is well established in 10^−13^ s.

Electronic polarization is present in all types of dielectric materials (>10^13^ Hz). It results from external electronic orbit displacement compared to the atomic nucleus. Electronic polarization is established in 10^−15^ s. The atomic, as well as electronic polarization, create dipoles that do not provoke energy loss and disappear as soon as the electric field is removed.

The different kinds of polarization that occur in a dielectric material under the influence of an electromagnetic field confirm that, in a protein (such as wheat gluten which can be considered as a polar material with amorphous and crystalline parts), polarization mechanisms are mainly of dipolar orientation (Debye type). In a frequency range, comprised between 100 MHz and 1 GHz, molecular chains, as well as amino acids are polarized.

### 2.2. Dielectric Properties of the Protein-Based Materials

In the process of film manufacture, vegetal (as wheat or corn gluten, soy, rape, and zein) and/or animal (collagen, gelatin, casein, ovalbumin, and keratin) proteins are available as an eco-friendly alternative. Gluten originates from albumen of cereals as wheat, zein are water insoluble storage proteins found in corn protein bodies [33], and collagen and gelatin come from pigskin. These protein based films can replace plastic packaging obtained from synthetic polymers due to their excellent film-forming properties. One of their main interests is based on their potential to provide special properties, such as some specific combination of water vapor, oxygen and carbon dioxide permeability values as flavor, aroma and oil barrier for food and pharmaceutical products to increase quality and shelf-life of the wrapped or coated products [34]. Edible films from proteins have already been considered for protecting food products considered as living tissues such as vegetables and fruits through carbon dioxide transfer control [13]. The partial elimination of carbon dioxide is of interest for maintaining products of high quality. Another application of gelatin and collagen films is for biomedical materials, e.g., the construction of devices that can replace defective tissues or organs (tissue implants, prostheses for nerve regeneration, and artificial skin) [35] or medicine delivery system [36].

Proteins are composed of a specific sequence of amino acids linked to each other by peptidic bonds. They possess a specific conformation, which influence their chemical reactivity and then their potential to create bonds which vary depending on localization, nature and/or energy [37]. Since proteins are constituted by polar repeating units of amino acid (–CO–CR–NH–) [11], and contain neutral, polar and charged side chains [38], they can be considered as polarizable materials. The three dimensional structures of these proteins are not well known and depend on many parameters such as origin, environmental conditions and extraction protocol. As an example, gelatin is formed by chemical hydrolysis of collagen cross-links. The amino acid composition of native collagen and gelatin prepared with an acid hydrolysis strongly differed [39]. In the case of alkaline prepared gelatin, the major part of fractions has a size around 100 kDa whereas gelatin obtained with an acid treatment exhibits a wide distribution of molecular weights. Due to the complexity of protein structures, others ways of characterization were developed. Four types of zein are classified (denoted α, β, γ and δ) according to their water solubility properties [33]. Gliadins and glutenins as the two main protein types of gluten are respectively soluble in a solution of 70% ethanol and diluted acid solutions.

Due to their polarisability, proteins are sensitive to dielectric techniques and present specific dielectric constant and loss factor in defined conditions (Table 1). The determination of dielectric properties of protein-based materials is currently used as a tool for material characterization [11,40].

The material processing parameters (plasticizer addition, pH, processing technology) and the environmental parameters such as water content, temperature and atmosphere composition affect the structure and/or composition of protein materials. Therefore, as dielectric properties of protein materials depend on their chemical composition and structure, material processing and environmental parameters influence the dielectric loss factor and the permittivity. The effects of various environmental and material processing parameters on dielectric characteristics are discussed in the next sections.

### 2.3. Frequency

As explained in the previous part, the polarization phenomena, and resulting dielectric constant and loss factor are frequency dependent parameters, considering the entire frequency range. The dependence of the dielectric permittivity and loss on frequency for protein films are represented in Figure 3. The occurrence of dielectric loss can be explained as follows: at lowest values of frequency, polarization follows the alternating field applied. This aspect implies that polarization contribution to dielectric constant is maximal and dielectric loss is neglected. At the highest frequencies, the field alternates rapidly resulting in the dropping out of slow polarization processes, thus decreasing the dielectric permittivity value. Wolf et al. [43] present dispersion regions within a protein solution, describing relaxation processes. The authors identify different peaks termed β, δ and γ, as a function of frequency, according to the biophysical nomenclature. β-relaxation occurs in the low frequency range and the γ-relaxation occurs in at higher frequency, around 18 GHz at room temperature. The authors assign respectively these relaxations to the rotation of polar segments of protein chains and to the reorientational motion of free water molecules, such as main relaxation processes in pure water. In the case of δ-relaxation, the complexity of proteins results in difficulties in deciding whether δ-relaxation can solely be explained by a bound water relaxation or if additional effects such as intra-protein motions have to be included. In addition to these descriptions taking into consideration only raw data, the authors also present and specify that the contribution of the β, δ and γ relaxations are dominated by dc conductivity at low frequencies [43].

Most of the literature studies reported on dielectric behavior of protein films were performed in a low frequency range, from 10^−2^ Hz to 1 MHz [11,35,40]. The authors were focused on the structure of the proteins particularly on the cross-linking in the films, associated to the relaxation processes and changes in the secondary structure. Furthermore, the denaturation of proteins and the hydration process of proteins were also studied to determine at which water content a change in behavior was recorded. Only few studies were performed at high frequencies: on wheat gluten powder on a frequency range between 200 MHz and 20,000 MHz [29], and on wheat gluten dough at 2000 MHz and 16,000 MHz [30] and 915 MHz and 2450 MHz [31]. Lima et al. [44] worked on collagen films in the frequency range of 100 Hz to 1800 MHz. There are also very sensitive and accurate capacitive methods for water content changing measurement with high resolution and high stability (in the frequency range between 1 MHz and 20 MHz). These capacitive sensing methods have high dielectric-to-frequency response, which increases significantly with relative humidity, and they are also temperature compensated [45]. In addition to this, these new and innovative methods reduce offset, temperature characteristic of main sensing element, temperature drift, and noise by a switching method, together with a fast dynamic response [46,47]. Nevertheless, at high precision capacitance or dielectric measurement, it is important to reduce any disturbing noise. The method developed by Bonfig [48] is a high precision capacitive method with very good noise compensation. It consists in using pseudo-stochastic excitation signals (de-convolution method), which very well compensate all kind of noise and temperature drift.

The permittivity values obtained by some of the authors are represented in Table 2. The high and low values of permittivity indicate that the dielectric technique is an effective tool for determining the amount of water content in the protein.

## 3. Factors Affecting Dielectric Properties of Proteins

### 3.1. Moisture Content

#### 3.1.1. Impact of Moisture Content on Dielectric Parameters

The dielectric properties of materials depend on their chemical composition, especially on the permanent dipole moments associated with water [49]. Water molecules access protein chains and form hydrogen bonds through their hydroxyl groups. As water percolates through the protein network, it accordingly changes the protein conformation and chain mobility.

As aforementioned, dielectric techniques are particularly sensitive to amount of moisture in samples and hence are excellent techniques to probe protein-water interaction and nature of bound water in biomolecules [41,50]. In particular, when water vapor is the sorbent, dielectric measurements are believed to be an effective tool for determining the macroscopic and microscopic state of water, because of its strong polarity.

Several studies testing protein films assessed by dielectric spectroscopy the dependence of the dielectric permittivity and loss on relative humidity (RH) conditions. Table 3 and Table 4 report typical values of ε′ and ε″ at low and medium relative humidity, obtained for different protein films.

Studies performed on the impact of relative humidity, e.g., water content, on dielectric parameters of protein films were assessed in a low frequency range (1–10^6^ Hz). At the lowest water content, when ε′ and ε″ values do not vary with frequency, no charge movement in the sample was indicated [40]. This aspect suggests that there are no charge displacements in this frequency range at low hydration level.

In the same frequency range when relative humidity rises, values of dielectric constant and loss factor increase and, as expected for an imposed relative humidity, both parameter values decrease with frequency augmentation. The curve shape (ε′ and ε″ vs. frequency) may be used as an indicator of the process of charge transport throughout the material.

The dielectric behavior of the majority of hydrated samples shows a dramatic increase of permittivity as frequency is decreased [51]. In the frequency range tested, this dielectric behavior is known as the low frequency dispersion (LFD) and occurs primarily in dielectric materials with large densities of low-mobility charge carriers. One subcategory of LFD is the quasi-dc process [52], in which the complex permittivity follows a fractional power law of frequency:

(2) $$\varepsilon^{*}=A^{*}\omega^{-p}$$

where A* is a complex constant and 0 < *p* < 1. If p is in range 0.8 < *p* < 1, then the LFD response may be assigned to the “hopping of charge carriers” between localized sites. In the case of hydrated protein, the protons are hopping within clusters of hydrogen bonds.

Quasi-dc processes are distinguished from dc-conductivity itself by the fact that the real permittivity of LFD is in parallel with the imaginary permittivity. It may be noted that the strong rise of ε′ at low frequencies in the case of quasi-dc processes implies a finite and reversible storage of charges in the material, which differentiates it from dc-conductivity in which there can be no charge storage, and consequently at low frequencies ε′ is independent of frequency. The fact that quasi-dc follows the power law of frequency and indicates that the LFD behavior was associated with a percolation process of charge carriers. For hydrated proteins, this means that protons percolated trough the protein matrix. The mobile protons originated from the ionizable groups of the protein molecule and the sorbed water molecules form hydrogen-bonded networks along which the protons can conduct. Authors using low frequency dielectric measurements to assess dielectric behavior of protein films [38,39,40,48] resorted to this theory to explain evolutions of dielectric parameters with frequency.

Water adsorption consists in the linking of initially isolated water molecules on the protein surface at the highest energy absorption sites. Absorption of water molecules then takes place throughout the volume of the protein layer. Therefore, additional water concentrates at these sites, forming clusters that continuously increase in size with water addition. Once clusters are increasing, several changes occur in the protein including protein conformation, internal protein mobility, and the mobility of water molecules.

An analysis of the literature data on hydration of gelatin enables to classify the sorbed water according to its state, into four types by nuclear magnetic resonance study [53]:

- **Water bound by high-energy sorption centers** (from 0 to 0.055 g·g^−1^ (0%–10% RH)). This type of water, existing at charged residues and inside the triple helix, plays a major role in protein stabilization, due the intramolecular hydrogen bonds.
- **Structural water** (from 0.055 to 0.14 g·g^−1^ (10%–40% RH)). This is the water directly bound to the protein (represented by both inside as outside helical fragments).
- **Polymolecular layer** (from 0.14 to 0.37 g·g^−1^ (40%–90% RH)). In excess of bound water concentration of 0.14 g·g^−1^, the monomolecular layer transforms into a polymolecular layer, which covers the triple helix structure.
- **Free water** (above 0.37 g·g^−1^ (90% RH)).

As the level of hydration increases in the protein film, there is an increase in the extent of charge movement and a change in the mechanism of charge transport. Almutawah et al. [40] reported that for gluten films, limited charge movement occurred below 8.6% water content. A change in the charge transport mechanism by charge-hopping occurred at water content of approximately 12%. At 43% water contained in the gluten sample, the Maxwell-Wagner behavior implies that surface hydration has reached a maximum level. Therefore, the “excess” free water directly guides the current through the electrodes. This Maxwell-Wagner behavior can be observed on the log-log evolution of ε″ plotted against frequency as in the higher frequency range (1 MHz). The same authors conclude that an increase in hydration level allows greater dielectric response, up to a point whereby a continuous channel of water is produced and a Maxwell-Wagner response due to the excess water is predominant. 

The reported studies in Table 3 and Table 4 were performed in the low frequency range and only one study was found on gluten dielectric properties determination at higher frequency range depending on relative humidity (200 MHz–20 GHz) [29]. In this frequency range, as soon as moisture content rises from 26% to 39%, the dielectric permittivity increases from 3 to 27 and loss factor exhibits a decreasing shape. As water content increases in wheat gluten samples, relaxation time slightly decreases, as a result of the increasing physical mobility of the mixture that becomes plasticized with water.

#### 3.1.2. Mechanical Properties of Protein Films

Typical mechanical characteristics of protein films such as elasticity modulus, tensile strength and elongation at break can be determined according to water content, in order to link structural and molecular modifications induced by hydration of the film macro-properties [54]. Cuq et al. [55] found that increasing water content caused a decrease in tensile strength and elasticity modulus with an increase in elongation of myofibrillar protein film.

Yakimets et al. [54] reported a mechanical behavior of gelatin films with respect to water content, which could be divided into three stages:

- Brittle behavior without any plastic deformation (0%–7% water);
- Brittle behavior with weak plastic deformation without decrease of cross-section area, which precedes the fracture (7%–14% water);
- Brittle behavior without any plastic deformation (14%–22% water).

Nevertheless, the fact that a brittle behavior without plastic deformation with 14%–22% water content is observed right after a brittle behavior with weak plastic deformation with 7%–14% water content has to be verified. The authors demonstrated the molecular, structural and macro-properties of gelatin films induced by the water content in a synthetic pattern. The latter clearly shows that coupling dielectric measurements to assess state of water in protein films with determination of mechanical, structural and molecular characteristics deepen the study of the material behavior with hydration content.

#### 3.1.3. Structural Consideration

To better understand structural and molecular changes occurring during hydration of protein films, dielectric measurements are often coupled to other analytical techniques, FTIR being one of them. Almutawah et al. [40] estimated that data obtained by both (FTIR and dielectric) techniques are complementary. According to their results, the infra-red data contained information that is essentially structural in nature. The changes in vibrational frequency represent changes in the electron distribution and density in the bonds involved in vibration. The time scale of the measurement is such that all states with lifetimes greater than 10^−13^ s contribute to the spectrum and are manifested in terms of the distribution of absorption frequencies observed. On the contrary, dielectric measurements are more sensitive to processes that take place between 7 and 15 orders of magnitude, than the vibrational processes. Changes in dielectric behavior will therefore reflect dynamical processes that are the result of changes in structure as observed by the infrared method. The combination of the two methods offers a route to understand the structure/function relationships arising from hydration.

**Figure 4:**
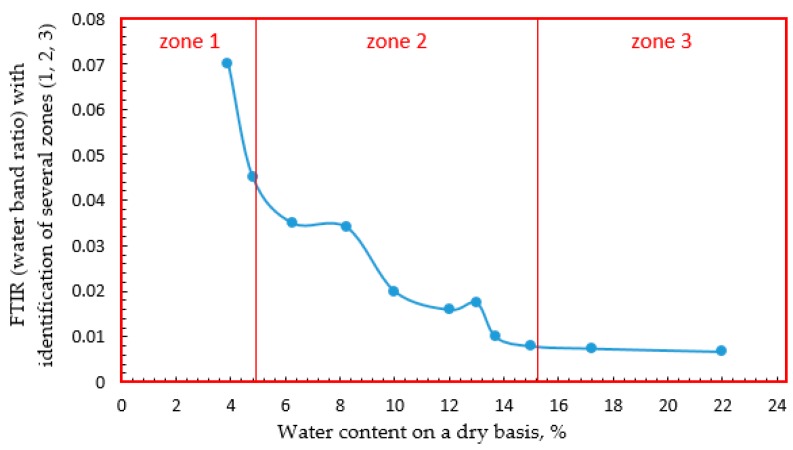
FTIR (Fourier transform infrared spectroscopy) water band ratio changes as a function of hydration. Three zones can be observed which correspond to: 1, high energy bonding of water to the protein; 2, steady medium rate of hydrogen bonding of water to the protein network; and 3, slow hydration rate of poly-molecular layer water to the modified network [54].

Yakimets et al. [54] have plotted the rate of the spectral changes observed on FTIR spectra in gelatin films as a function of water content (Figure 4) and revealed different regions that could be associated with the different types of hydration.

For water content up to 5%, water molecules bind to the protein network with a high energy. This high energy sorption effect could be observed by the high initial rates of changes in the infrared spectra for the O-H band of water and the amide bands of the protein (zone 1). Between 5% and 15% water content, spectroscopy showed a steady medium rate increase in hydrogen bonding of water to protein, which corresponded to the medium water activity (zone 2). Above this point, the hydration rate slowed down significantly, as well as the spectral changes in the protein. Between 15% and 22% the amide bands continued to change slowly as polymolecular layer water was added, and above 22%, the spectra remained constant, indicating that the protein was fully hydrated and did not sense any additional water (zone 3). Almutawah et al. [40] also observed for wheat gluten samples an evolution of amide II band depending on water content. They reported that between 30% and 40% water content there is a significant change in the environment of N-H groups becoming fully hydrated above this water content percentage, and their hydrogen bonding environment changes only slowly from that point. This is compatible with the results of Belton et al. [56] who noticed that a large change in the shape of the amide II band occurred at about 30%–37% water in high molecular weight subunit of glutenin.

Kusanagi and Yukawa [57] reported that FTIR is one of the effective techniques to study the intermolecular interactions between various solid polymers and water molecules, which are sorbed within them. Authors used the water affinity parameter (WAP) estimated using the shift in the stretching of hydrogen bonds of the sorbed water molecule (liquid or gaseous) and they reported the reliable indication given by WAP of the site affinity to water molecules in the polymer. Using this parameter, it is possible to clarify the water structure in the solid polymer medium, e.g., the cluster formation propensity of the sorbed water. Judging from the definition, if the state of the sorbed water is close to that of liquid water (clustered state), the WAP values become nearly equal to unity. On the contrary, if the state of water molecule is close to that of the vapor (isolated state), a parameter value near zero is obtained.

#### 3.1.4. Alternative Method for Molecular Consideration

Infra-red spectroscopy is an important technique for the structural study of materials at the molecular level and has also been applied for understanding the structure of sorbed water molecules in polymers. Unfortunately, none of these techniques can provide suitable information related to polarity and/or hydrogen-bond acidity of polymers. Matsuguchi et al. [58] applied an alternative way to the characterization of solid polymer: the solvatochromic method. They prepared thin polymer cellulosic or synthetic films containing certain media sensitive dyes and measured the UV-Visible absorption spectra of the films. According to the authors, the measured empirical parameters constitute a more comprehensive measure of the characteristics, especially for polarity and/or the hydrogen bonding ability (e.g., donor (hydrogen bonding acidity) and acceptor (hydrogen bonding basicity)) of polymers than the dielectric constant determination or any other single physical characteristic, since they reflect with more accuracy the complete picture of all intermolecular forces. Matsuguchi et al. [58] also confirmed that this method is adapted to understand the interaction of polymer with gaseous water molecule.

According to Paley et al. [59], three solvatochromic parameters are defined, e.g., dipolarity/polarizability (π*_1_), hydrogen bond acidity and basicity respectively named as α and β. Matsuguchi et al. [58] proved that hydrophilic polymers presented high value of π*_1_ and β, implying that these polymers have strong sites able to accept hydrogen bonding formation with water. These three parameters are affected by relative humidity. As π*_1_ and α slightly increased to values obtained in bulk water with an augmentation of relative humidity, β value first increased and then decreased as soon as the cluster formation started, e.g., β parameter is effective for the elucidation of the state of sorbed water in polymer films. It is the case for ethyl-cellulose film. First with the elevation of relative humidity, hydrogen bonding basicity increased. This means that the sorbed water in polymer film was changed from a gaseous state to a liquid one with increasing amount of sorbed water. These polymers have hydrogen bonding basic sites. The hydrogen bonded water molecules by basic sites become acidic sites, which bind to the basic O site of the water molecule. As a result, the cluster formation is enhanced.

### 3.2. Other Plasticizer Content than Water Content

#### 3.2.1. Plasticizer Content Effect on Dielectric Parameters Evolution

Plasticizers are small molecules of low volatility which once added to protein polymeric materials modify the three dimensional structure, decrease attractive intermolecular forces and increase free volume and chain mobility. These molecules such as glycerol are small enough to insert between protein chains and to form hydrogen bonds through their hydroxyl groups [60]. This molecule type is used to avoid film brittleness resulting from extensive intermolecular associations. Content of plasticizer added to protein film results in structural and physical changes of polymer films. Dielectric properties are influenced by plasticizer content. Some authors assessed the evolution of dielectric properties of gelatin [61,62,63] and zein films [42] depending on plasticizer content.

With zein film, Gillgren et al. [42] showed that at the same frequency of 1 MHz, increasing glycerol content from 24% to 40% resulted in a slight augmentation of loss factor whereas dielectric constant were the same. If frequency was lowered to 0.01 Hz, both dielectric parameters exhibit significant increase with glycerol content as at these low frequencies where the different types of polarization occurred. Increase of dielectric constant with glycerol content was also observed by Bergo et al. [63] who worked with gelatin films.

Different kinds of plasticizers can be used such as glycerol, 2-mercaptoethanol, ethyleneglycol, diethyleneglycol and polypropyleneglycol, other than water. Glycerol is a relatively small polar molecule that has been widely used as a plasticizer and may have similar mechanisms of plasticization as water molecules. 2-mercaptoethanol is less polar but is responsible for breakage of disulfide bonds which affect the internal mobility of proteins. Thus it may be an alternate form of plasticization as proposed by Gillgren et al. [42].

#### 3.2.2. Effect of Plasticizer on Mechanical Properties of Protein Films and FTIR Spectra

Plasticizer content affects mechanical properties of protein film samples [62,64]. Bergo et al. [63] found that tensile strength at break and elasticity modulus of gelatin films decreased as glycerol content increased from 0% to 15%, 30% and 45%. In the same time, elongation at break rises with glycerol content. The plasticizer weakened the intermolecular forces between the chains, reducing the stress at rupture, increasing the mobility between the gelatin chains in the matrix, which gives more flexibility to the film.

The same authors examined the interactions of zein film and plasticizer at the molecular level. Currently, little is known about the molecular details of the plasticization process, and Gillgren et al. [42] recommend obtaining more information about the molecular processes involved during plasticization. They reported plasticizing effects of water and glycerol by a combination of spectroscopy FTIR, dielectric spectroscopy and thermomechanical methods based on dynamic mechanical analysis (DMA). Their FTIR and dielectric spectroscopy results exhibited the similarity of the plasticizing effects of water and glycerol.

Indeed, in samples with 12.3% water content and 40% glycerol, the amide II band of both samples has a reduced intensity at 1515 cm^−1^, compared with that of the dry material. Generally, the amide II band responds to differences in the hydrogen bonding environment [40]. Thus, authors presumed that both samples have similar effects on the hydrogen bonding of the amide groups and that amide-amide interactions are reduced by an increase of amide-plasticizer interactions. The amide I band changes are similar in size to the amide II band changes. The dry material shows a broader peak with the main intensity around 1650 cm^−1^, consistent with a α-helical structure. Bands observed at 1620 cm^−1^ and 1680 cm^−1^ are probably both due to sheet-like structure [65]. In the case of glycerol sample, the intensity of bands due to sheets is diminished. These results seem to indicate that glycerol enhances the formation of α-helical forms. The sample with water, on the other hand, while reducing the 1680 cm^−1^ band intensity, mildly enhances the 1620 cm^−1^ band intensity. The authors suggest that this may be due to differences in the detail of hydrogen bonding patterns and other factors such as the differences of dielectric constant of water and glycerol respectively of 78.5 and 42.5 [66] at ambient pressure and temperature.

A model consistent with the data is that separate plasticizer molecules are first adsorbed on the surface of zein proteins. Thereafter, clusters grow around these sites as more plasticizer molecules bind to the protein. The lack of bulk plasticizer indicates that the binding sites are not fully occupied at the maximum concentrations (e.g., water content from 0% to 12.3% and glycerol content from 0% to 16%) of plasticizer used in their study.

Plasticizer effect on physical and structural protein film properties are often related to moisture content. It is the case of Bergo et al. [61] who assessed that the gelatin films containing glycerol presented increased water absorption in comparison to films containing ethyleneglycol, diethyleneglycol and polypropyleneglycol. Indeed, some plasticizers such as glycerol contain hydrophilic groups, where the water molecules can be linked by hydrogen bonds, resulting in strong moisture absorption [67]. Thus, some publications try to relate the interactions between plasticizer content, film structure and moisture content.

Bergo et al. [63] worked on the dielectric properties of gelatin films as a function of glycerol content at 0%, 15%, 30% and 45% and moisture loss. For this test, the films were previously conditioned in silica gel, for different periods of time (7, 14 and 21 days). The dielectric constant decreased with moisture loss and increased with glycerol content. At storage periods greater than 14 days, the dielectric behavior of each film seems to reach an equilibrium value, but still increases with glycerol content. This can be assigned to the contribution from glycerol in the film structure and some residual bound water in the film, to the total polarizability. The authors also analyzed the structural effect of storage at different RH of gelatin samples with and without glycerol. Samples were conditioned for a week in three different salts (silica, P_2_O_5_ with RH below 10% and NaBr with RH 65%). Structural effect was assessed by comparing the variations in the position of the amide II peak of FTIR spectra. The large variations in the position of the amide II peak were observed for those films conditioned in a very low moisture environment containing P_2_O_5_, and without plasticizer, which can be due to the dominance of bound water and strong O–H bridges in the film matrix. With moisture content increasing (due to plasticizer and RH), the variations in the amide II peak position become less prominent, reaching an equilibrium position for films containing 45% glycerol, possibly due to the increase in free water content occupying interstitial positions in the polymeric chains [54]. Therefore, the position of amide peaks depends on the interaction type of water molecules with the film structure.

### 3.3. Temperature

#### 3.3.1. Temperature Dependence of Dielectric Parameters

Several studies relate the evolution of dielectric parameters with temperature [35,36,41]. The curves representing ε′ and ε″ with temperature allow accessing different structural information.

**Figure 5:**
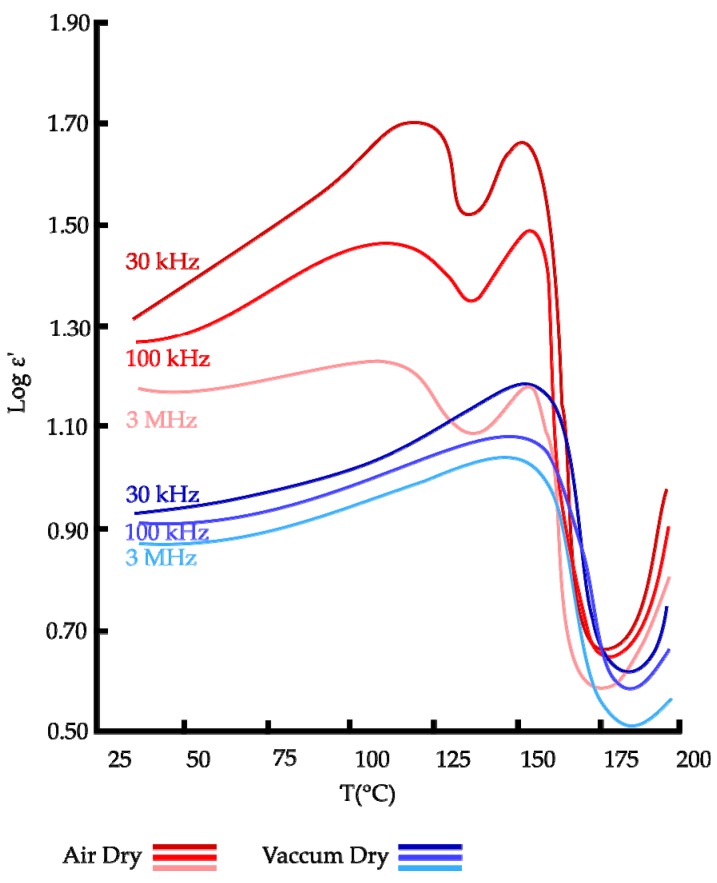
Temperature dependence of dielectric constant for air and vacuum dry keratin [41].

Rizvi and Khan [41] plotted the temperature dependence (30 °C to 200 °C) of dielectric constant for three different frequencies (30 kHz, 100 kHz and 3 MHz) of keratin films in air (RH of 50%) or dry vacuum (RH of 0%). With this method, authors accessed to the type of binding between water and keratin polymer for samples either in dry air or dry vacuum. A typical curve obtained in their study is presented in Figure 5. At a first glimpse, at low frequencies (30 kHz), conductivity dominates and polarizes electrode-protein interfaces. Interfacial polarization arises when the conducting ions in the sample arrive at the metallic electrodes and accumulate in thin layers immediately beneath the sample forming a space-charge region. This affects the dielectric spectra of any material containing free ions. As frequency rises, the conductivity decreases as ions are no longer able to follow the quick changing electric field. As such, the peaks observed at low frequency (30 kHz) in Figure 5 tend to disappear at higher frequencies (100 kHz and 3 MHz). On the other hand, DSC analysis could be performed to prove these phenomena.

The peak observed only at 120 °C on dry air sample was due to the presence of loosely bound water in the sample. At 155 °C, the peak observed in both kinds of samples is relative to strongly bound water that partially remained in the dry vacuum sample after 72 h in a vacuum oven at 105 °C. Authors reported that the presence of two types of water bound exhibiting two distinct peaks in the temperature dependence of dielectric constant, can be assessed in terms of two kinds of binding sites in keratin molecules. As keratin is composed of amorphous and crystalline regions with crystalline sites embedded inside amorphous regions, authors suggested that the exposed amorphous regions provide weakly bound water sites whereas the internal crystalline regions provide strongly bound water sites.

The study also explained the dielectric constant behavior with the increase of temperature. From 30 °C to 120 °C a net augmentation of ε′ was measured due to the increase in cluster size around polar sites of the keratin and hence the average separation between protons sites, which results from increased amplitude of vibrations of water molecules. The peak present at 120 °C indicates a maximum change in cluster size around polar protein components in the amorphous region at this temperature. Above 120 °C, water molecules bound to the polar sites in amorphous region acquire sufficient energy to overcome their binding energies. This aspect result in the release of bound water from the amorphous region which causes a decrease in the number of proton sites for charge percolation in the amorphous region and hence, a reduction of dipoles number causing a decrease of permittivity. When temperatures get even higher, a different peak is observed at 155 °C. It is attributed to the increase in cluster size around polar sites of the keratin in crystalline regions. After this peak, permittivity decreases because almost all water is driven out of keratin (the free or strongly bound water of, respectively, amorphous or crystalline region). Above 175 °C, dielectric constant increases again which is attributed to the onset of chain melting in the crystalline region.

#### 3.3.2. Dielectric Parameters and Glass Transition Temperature

The dielectric relaxation curves allow determining chain movements by comparison at different temperatures and different frequencies. These curves also permit to access typical positions as vitreous transition characterized by glass transition temperature (T_g_) which is one of the most important properties of polymers directly related to structure modifications.

It is the case of dielectric thermal analysis (DETA) and dynamic mechanical analysis (DMA), which collect data of dielectric constant and loss factor at a specific heating rate. As an example, Hochstetter et al. [68] used DETA technique on gluten film at a heating rate of 2 °C·min^−1^ from −150 °C to 50 °C. T_g_ is assessed as a peak temperature of tan δ (ε″/ε′) at 100 Hz. Other techniques such as differential scanning calorimetry (DSC) can be used [36,44,54]. Evolution of T_g_ is then assessed on protein film depending on relative humidity conditions [36,68], water [54], plasticizer content as glycerol [42], frequency [68] and cross linking agent addition [11].

On gluten films, T_g_ decreased from −53 °C to −79 °C with increasing RH from 0% to 77% due to plasticization effect of water [68]. The same authors used the Arrhenius equation to model relationship between the frequency used and T_g_ observed. They identified the shift of T_g_ to lower values with increasing storage RH. The results obtained by Yakimets et al. [54] on T_g_ evolution of gelatine films with water content matched the ones of Hochstetter et al. [69] on gluten films. Their T_g_ clearly decreases from 120 °C to around 30 °C as water content reached 3% to 25% in samples.

Concerning T_g_ evolution with glycerol content, Gillgren et al. [42] showed the decrease of T_g_ of zein film from 160 °C to 50 °C as glycerol content is increased from 0% to 40%. They also assessed water as a more efficient plasticizer than glycerol as it took approximately 8% water content to reduce T_g_ from 160 °C to 60 °C, whereas it required 24% glycerol to have the same effect. Al-Hassan and Norziah [64] also reported the decrease of T_g_ of starch-gelatin films with the addition of glycerol.

#### 3.3.3. Impact of Temperature on Protein Films at Molecular Level

As dielectric parameters evolution with temperature (or others parameters) are not completely clear, some authors tried to study protein films in a molecular point of view as Georget et al. [65] who evaluated the effects of temperature (25–85 °C) on gluten secondary structure at different hydration levels (0%, 13% and 47%), thanks to FTIR spectroscopy. At 0% hydration, no change in the secondary structure with temperature was detected. The spectra were consistent with a tight disordered structure with many protein-protein interactions. At 13% hydration, distinctive changes occurred in the low frequency region of the amide band I (1630–1613 cm^−1^). This was attributed to changes in the β-sheet structure. On cooling to 25 °C, these changes were mainly reversed. It was observed that most of the changes occurred above the glass transition temperature (45–55 °C). At 47% hydration, more complex changes took place: as the temperature was raised, distinct bands at 1630 cm^−1^ and 1613 cm^−1^ merged. However, this process was partially reversed, with recovery of both bands, on cooling.

### 3.4. Cross-Linking Agent

Crosslink is the covalent bonding of one polymer chain to another, forming protein-protein interactions. Some protein films such as collagen ones used as biomaterials in medicine field can require specific surface configurations, surface chemical modification, pharmacologically active surfaces and/or biological integration depending on the biomedical requirements [35]. In that way current researches try to develop collagen films with cross linking agents such as glutaraldehyde [11], chitosan [44], chondroitin sulfate or hyaluronic acid [35,69]. These studies assessed the influence of cross linking agent concentration on dielectric constant and dielectric loss.

Dielectric constant shows typical decrease with the increase of frequency from 10^−2^ Hz and 10^6^ Hz for collagen film with glutaraldehyde [44] or with chitosan from 1 MHz to 1 GHz [44]. With glutaraldehyde, the dielectric permittivity is found to decrease with increasing crosslink at lower frequencies and attains a constant value at higher frequencies. The effect of crosslink was to decrease and stabilize the dielectric constant at lower frequencies, which can be related with the lower molecular mobility due to the cross linking process.

Concerning the influence of cross-linking agent on dielectric loss evolution with frequency, Figueiro et al. [11] proved the existence of a shift of ε″ peak with frequency, with an increase in crosslink. These peaks are consistent with a relaxation process occurring in the collagen films. Thus, the dielectric relaxation is sensitive to the motion of charged species and dipoles of the macromolecules in the films.

Marzec and Pietrucha [35] found a method based on a combination of thermal and vacuum drying and a chemical treatment cross-linking, which allows to significantly increase values of ε′ and ε″ in a temperature range of 20 °C to 240 °C and in a low frequency range. The authors assumed that this cross-linking method allowed obtaining a greater number of charge carriers and sites available in order to accumulate and release current.

### 3.5. Gas and Volatile Content

Polymer films such as protein films exhibit different behaviors to gas transfer. Their study also raised the interest, as the purpose of this review is to assess the effects on dielectric parameter values of protein (such as gluten) films, in the presence of gas such as carbon dioxide (CO_2_), oxygen (O_2_), ethanol and ammonia. Polymer films permeability represents the product of the diffusion coefficient, representing the mobility of permeate molecules in the polymer, and of the solubility coefficient, representing the permeate concentration in the film in equilibrium with external pressure. Temperature, shape, size and polarity of the diffusing molecules affect the diffusion and solubility of permeates. Furthermore, solubility and diffusion are affected by film characteristics, including the type of forces influencing molecules of the film matrix, the extent of cross linking, the degree of crystallinity and the presence of additives such a plasticizers [60].

The O_2_ and CO_2_ permeability values of wheat gluten films are highly dependent on RH and temperature. At low RH and temperature, the O_2_ and CO_2_ permeability values are low compared to values obtained at higher temperature and RH. For example, Mujica-Paz and Gontard [14] found that when RH was increased from 0% to 100% at 24 °C, CO_2_ permeability increased from 88 amol·m^−1^·s^−1^·Pa^−1^ to 55.580 amol·m^−1^·s^−1^·Pa^−1^ and O_2_ permeability increased from 77 amol·m^−1^·s^−1^·Pa^−1^ to 1970 amol·m^−1^·s^−1^·Pa^−1^. Hochstetter et al. [68] also found an increase of oxygen permeability of gluten film as RH increases from 0% to 50%. For these gases, permeability remains low when RH does not exceed 50%, regardless of the temperature, and rose steeply at RH over 50%, where for CO_2_, permeability increase is higher than for O_2_. This result could be explained by the difference in water solubility of both gases, implying that CO_2_ is highly water soluble but may also be explained by specific interactions between CO_2_ and the water plasticized protein matrix.

Concerning ethanol, the only relevant interesting result from literature does not concern ethanol vapor but the addition of ethanol as solvent during natural polymer film preparation [69] and/or as dissolution medium [70]. Ethanol is added to evaluate its influence on water permeability. Zein-oleic film formulation was done with 75%, 80%, 85%, 90% and 95% (*v*/*v*) of ethanol as zein solvent. Water vapor permeability is lower when films were produced with higher ethanol concentration. The authors reported that the increased ethanol concentration allowed the formation of higher intermolecular forces between protein chains, preventing the water vapor migration through the film [69]. When ethanol was added from 0% to 40% (*v*/*v*) as dissolution medium to cellulose film [71], water permeability increases.

Only one study was found on typical behavior of dielectric parameters of wheat gluten film deposited on designed and manufactured interdigital capacitor jointly with CO_2_ and ethanol contents [72]. The purpose of this study was to observe the impact of these two typical volatile markers of food freshness on dielectric properties of wheat gluten for further development as bio-sensors in food packaging. At 80% relative humidity and 25 °C, the dielectric permittivity of hydrated wheat gluten proteins increased from 6.50 ± 0.07 up to 10.71 ± 0.02 when CO_2_ concentration rose from 0% to 40% and up to 12.75 ± 0.03 when ethanol concentration rose from 0% to 0.1%. Authors mentioned that these effects of CO_2_ and ethanol vapor could be linked to the numerous possible interactions of these molecules with wheat gluten proteins as suggested in Pochat-Bohatier et al. [73] such as hydrogen binding with polar amino acids, e.g., glutamine, hydro-gen bindings with peptidic chains and/or hydrophobic interactions with hydrophobic lateral groups of the constituting amino acids.

## 4. Potential Development of Proteins as Biosensors

For at least 20 years, synthetic polymers have been widely used in the field of electronic measuring devices such as sensors. Semi conductors, semi conducting metal oxide, solid electrolytes and ionic membranes have been the current materials employed for sensor design [74]. Those polymers were developed as humidity, gas or pH sensors among others applications. Two decades ago, the use of proteins as sensor device has remained minime and has consisted on protein film as keratin or bovine plasma albumin crosslinked with glutaraldehyde immobilized on a quartz crystal microbalance sensor for selective adsorption of a pesticide (strychnine) and an odor (β-ionene) [74]. The relation between dielectric properties of proteins and environmental changes was recently taken into account to develop a humidity sensor. Bibi et al. [75] investigated a thin wheat gluten protein film deposited on designed and manufactured interdigital capacitor as humidity sensor in food packaging on the frequency range from 30 MHz to 1000 MHz. When relative humidity increases from 20% to 95% RH at 25 °C, authors showed dielectric permittivity and loss change from 5.01 ± 0.04 to 9.79 ± 0.06 and from 0.39 ± 0.01 to 1.48 ± 0.02, respectively. The dielectric permittivity and loss were very sensitive to relative humidity and increased exponentially due to the comparable exponential increase of wheat gluten water content versus relative humidity. Authors attested that the dependency of both dielectric permittivity and dielectric loss of wheat gluten on relative humidity offered the possibility to use the protein for monitoring relative humidity in packed food products.

Bibi et al. [75] compared the capacitance sensitivity and the hysteresis of different humidity sensors found in literature with their device designed with wheat gluten. The capacitance sensitivity was defined as:

(3) $$s=\left(\frac{C^{u}-C^{i}}{X^{u}-X^{i}}\right)$$

where *S* represents the sensitivity of wheat gluten coated IDC, *C* represents the capacitance value, *X* represents the concentration of relative humidity, *u* represents the final values and *i* the initial values.

The maximum hysteresis was calculated as:

(4) $$Max\;Hysteresis=\frac{max|C_{dec}-C_{inc}|}{C_{max}-C_{min}}\times 100\%$$

where *C_dec_* represents the decreasing capacitance value, *C_inc_* the increasing capacitance value, and *C*_max_ and *C*_min_ the maximum and minimum capacitance values, respectively.

Table 5 shows the characteristics of humidity sensors. Listed are the sensitivity and hysteresis of a natural polymer, synthetic polymers and oxide based polymers where most of them have been studied to be coupled to electronic measuring devices. Although some materials (Cerium (IV) oxide, and porous aluminum oxide) have high sensitivity performances, wheat gluten has a very good sensitivity value on a very wide range of relative humidity, and has a major advantage over the other materials being a natural polymer and a by-product of the agrifood industry. This ensures a much lower cost than the others. In addition to this, wheat gluten has a very low hysteresis indicating that the measurements are reversible on several relative humidity (RH) sorption and desorption cycles, which is not the case of the other materials (except Cerium (IV) oxide).

As mentioned in previous section, impact of carbon dioxide and ethanol vapor on wheat gluten film dielectric properties and coated on electronic device for bio-sensor of food freshness was investigated [72]. Authors affirmed that results obtained appeared of interest in the field of food marker sensors, but need to be deeply investigated for further development.

## 5. Conclusions

The present review gives an overview of the dielectric properties of natural polymers, proteins for instance. Several aspects are put forward such as the dielectric dispersions that correspond to different types of polarization in the material, which are frequency dependent, and the dependency of the protein structure on moisture content, plasticizer content and temperature. The mechanical properties, as well as structural characterization techniques such as infrared spectroscopy (FTIR) and glass transition temperature determination (DETA and DMA) using dielectric spectroscopy were reviewed and presented.

The evolution of the dielectric constant and dielectric loss factor of protein films has been mainly studied at low frequency (10^−2^ to 10^6^ Hz). Few literature references evaluate the dielectric parameters of protein films at high frequency. It would be of great interest to study proteins at high frequency as a function of different analytes, in terms of dielectric characterization, and, simultaneously, assess mechanical properties (tension test), thermal and structural characteristics and vibrational properties, to determine the subsequent changes on the protein structure.

This investigation on the relation between the dielectric properties of proteins and environmental parameters was necessary for a better understanding of the promising potential use of proteins as bio-sensors. Proteins are thought to interact with the so-called markers of food degradation, such as water vapor, oxygen, carbon dioxide, ammonia and ethanol commonly associated to extensive and detrimental microbial degradation or respiratory activity. The development of a natural polymer could be an innovation in the bio-sensor technology. In spite of its complexity, it would have significant financial and environmental potential for bio-sensor development, assuming that detection of fluctuations in dielectric properties, influenced by changes in environmental conditions, is possible. These changes in dielectric properties could be furthermore intelligently modulated thanks to adequate plasticizers and/or nano-particles. It is concluded that the present review opens new perspectives for intelligent packaging due to the potential development of innovative, eco-friendly bio-sensors from natural polymers.

## Figures and Tables

**Table 1:** Typical values of ε′ and ε″ for dry natural polymer films (e.g., low relative humidity below 10%).

Protein Type	Frequency	Temperature	ε′	ε″	Reference
Gelatin	1 MHz	25	12	0.2	[11]
Gluten	1 MHz	15	7	0.08	[40]
Keratin	3 MHz	50	8	0.2	[41]
Zein	1 MHz	25	7	8.10^−2^	[42]

**Table 2:** Dielectric permittivity and loss of some proteins found in literature as a function of parameters such as frequency and water content.

Material	Method Used	Frequency	% Relative Humidity (T/°C)	% Water Content (Dry Basis)	ε′	ε″	Ref.
Gluten (Dough)	Open ended coaxial cable connected to a probe	915 MHz	- (30)	92.3	27.3	8.6	[31]
		2450 MHz	- (30)	92.3	24.2	8.1	
Gluten (Dough)	Open ended coaxial line probe	2000 MHz	- (22)	38.8	5	2.5	[30]
		16,000 MHz	- (22)	38.8	3.5	2.25	
Gluten (powder)	Open ended coaxial line probe	200 MHz	- (22)	63.9	22	79.5	[29]
		10,000 MHz	- (22)	63.9	12.5	7.95	
		20,000 MHz	- (22)	38.8	3.5	0.79	
		3 MHz	~0 (50)	-	15.8	2	
Collagen (films)	-	1 MHz	-	-	3.94	-	[44]
		1000 MHz	-	-	2.71	-	

**Table 3:** Dielectric permittivity and loss values of protein films at low relative humidity (<50% RH).

Material	Frequency Range (Hz)	Temperature (°C)	ε′	ε″	Reference
Keratin	30–3 × 10^6^	50	10–8	2–0.2	[41]
Gluten	10^−2^–10^6^	15	7	0.1–0.08	[40]
Zein	10^−2^–10^6^	25	7–5	1–0.1	[42]

**Table 4:** Dielectric permittivity and loss values of protein films at medium relative humidity (50%–84% RH).

Material	Frequency Range (Hz)	Temperature (°C)	Relative Humidity (%)	ε′	ε″	Reference
Keratin	30–3 × 10^6^	50	50	250–13	100–2	[41]
Gluten	10^−2^–10^6^	15	60	10^8^–10^2^	10^8^–10^3^	[40]
Zein	10^−2^–10^6^	25	84	10^6^–10	10^6^–1	[42]

**Table 5:** Humidity sensor characteristics found in literature [75].

Material	Type	Max. Sensitivity	Max. Hysteresis	Application
Wheat gluten (film) [75]	Natural polymer	162.0 ± 0.6 fF/%RH between 90% and 95% RH	7% at 90% RH	Agrifood sector-Passive RFID
Anodic aluminum oxide [76]	Oxide	483 fF/%RH	30% at 92% RH	Humidity sensors
Polyethylene-naphthalate and polyimide foils [77]	Synthetic polymer	21 fF/%RH	-	Intelligent RFID
Polyimide [78]	Synthetic polymer	4.5 fF/%RH	10% at 50% RH	Humidity sensor for RFID for monitoring environmental humidity
Cellulose acetate butyrate [79]	Synthetic polymer	1.5 ± 0.03 fF/%RH	20% at 20% RH and 30% RH	Low cost sensor arrays and disposable sensing platforms
Porous aluminum oxide [80]	Oxide	312.5 fF/%RH	-	-
Polyphenylacetylene (PPA) [81]	Synthetic polymer	10 fF/%RH	-	-
Anodic Aluminum oxide [82]	Oxide	4200 fF/%RH	16.5% at 70% RH	Humidity sensors
Cerium(IV) oxide [83]	Oxide	111 pF/%RH	1% at 45%RH	Humidity sensors
